# Differential Oxidative Stress Profiles in Circulating and Peritumoral Adipose Tissue Across Stages of Colorectal Cancer

**DOI:** 10.3390/ijms27020707

**Published:** 2026-01-10

**Authors:** Somchai Ruangwannasak, Sittichai Khamsai, Poungrat Pakdeechote, Putcharawipa Maneesai, Parichat Prachaney, Wilaiwan Mothong, Chalerm Eurboonyanun

**Affiliations:** 1 Department of Surgery, Faculty of Medicine, Khon Kaen University, Khon Kaen 40002, Thailand; somcru@kku.ac.th; 2 Department of Medicine, Faculty of Medicine, Khon Kaen University, Khon Kaen 40002, Thailand; sittikh@kku.ac.th; 3 Department of Physiology, Faculty of Medicine, Khon Kaen University, Khon Kaen 40002, Thailand; ppoung@kku.ac.th (P.P.); putcma@kku.ac.th (P.M.); 4 Department of Anatomy, Faculty of Medicine, Khon Kaen University, Khon Kaen 40002, Thailand; parpra@kku.ac.th (P.P.);

**Keywords:** colorectal cancer, malondialdehyde, protein carbonyl, catalase activity, superoxide dismutase activity

## Abstract

This study intends to assess oxidative stress markers and endogenous enzymes in plasma and peritumoral adipose tissues (PATs) obtained from normal subjects and patients with stages I-IV colorectal cancer (CRC). 63 participants were recruited, including 23 patients with colorectal cancer and 40 healthy subjects. CRC patients had increased circulating malondialdehyde (MDA) and protein carbonyl concentrations, as well as reduced superoxide dismutase (SOD) and catalase activities, compared to normal volunteers. (*p* < 0.05). The findings aligned with the oxidative parameters assessed in peritumoral adipose tissue. Superoxide production in PAT was dramatically higher in the CRC group compared to the control group (*p* < 0.05). Moreover, oxidative stress markers were progressively altered in relation to CRC stages. Nuclear factor erythroid 2-related factor 2 (Nrf2)/heme oxygenase-1 (HO-1) protein expression was reduced in PAT isolated from CRC compared to normal subjects and associated with CRC stages. CRC patients showed a systemic and peritumoral oxidative imbalance, along with elevated superoxide production in the PAT. The oxidative modifications worsened with the progression of CRC stage and were associated with the downregulation of the Nrf2/HO-1 antioxidant cascade in peritumoral adipose tissue.

## 1. Introduction

Colorectal cancer (CRC) refers to benign tumors of the colon and rectum. It is the third most common cancer type and the leading cause of global mortality [1]. The precise cause of colorectal cancer remains unclear; nevertheless, environmental, genetic, dietary, smoking, and lifestyle variables enhance its incidence [2]. Data on the incidence rate and new cases of CRC have climbed to 3.2 million and 1.6 million fatalities in 2040, with the majority of cases expected to occur in countries with high or very high Human Development Index values [3]. According to reports, the survival rate of CRC is determined by the stage of the disease, as the survival rate decreases with each stage: I, II, III, and IV [4]. The five-year survival rate is abysmal in metastatic CRC [5]. The pathogenesis of CRC involves various factors, with oxidative stress suggested as a possible initiator and contributor to CRC progression [6].

Reactive oxygen species (ROS), such as superoxide, hydroxyl radicals, and hydrogen peroxide, can be mitigated by superoxide dismutase and catalase, thus maintaining cellular homeostasis [7,8]. The nuclear factor erythroid 2-related factor 2 (Nrf2)/heme oxygenase-1 (HO-1) cascade plays an essential role in modulating defenses against oxidative stress. Nrf2, a basic leucine zipper transcription regulator, is indispensable for controlling cellular antioxidant responses and triggers several antioxidative proteins [9,10]. Oxidative stress arises from an overabundance of ROS coupled with insufficient antioxidant defenses. ROS Under typical physiological conditions, low levels of reactive oxygen species (ROS) are a normal aspect of cellular function with regulation of various signal transduction pathways; however, elevated levels of ROS can damage cellular function and structure, including lipids, proteins, and nucleic acid components, leading to cell death processes, apoptosis and cancer [11,12,13]. Consequently, numerous investigations have demonstrated elevated levels of oxidative stress biomarkers, including malondialdehyde (indicative of lipid peroxidation), 8-oxo-2’-deoxyguanosine (signifying DNA oxidation) and protein carbonyl (reflecting protein oxidation) in cancer cases [14,15]. Evidence indicates that CRC patients exhibit elevated levels of circulating (plasma) malondialdehyde (MDA), which correlates with the stages of CRC [15].

Growing interest now focuses on the tumor-adjacent stroma and its role in driving progression. The peritumoral microenvironment, encompassing the extracellular matrix, fibroblasts, adipocytes, endothelial cells, and immune cells, actively modulates tumor dynamics and evolves in parallel with cancer cells [16]. Adipose tissue near developing tumor cells might intensify this pro-oxidant environment by releasing inflammatory cytokines that enhance both inflammation and oxidative stress, thereby facilitating cancer cell growth [17]. According to Conti and coworkers (2013), adipose tissue adjacent to primary lesions and nodal metastases in a colorectal cancer preclinical model exhibits mesenchymalization over the course of tumor progression [18]. Evidence in CRC cells and male homozygous athymic mice showed that peritumoral adipose tissue (PAT) promoted metastases. However, limited information on oxidative stress markers and the antioxidant defense system in peritumoral adipose tissue during colorectal cancer staging has been reported. We aim to assess the significance of oxidative stress, antioxidant biomarkers, and endogenous antioxidant enzymes at different stages of colorectal cancer in patients. This study demonstrates that plasma and PAT from CRC patients show a stage-dependent elevation in oxidative stress markers, alongside a corresponding deterioration of essential antioxidant defenses. Furthermore, oxidative and antioxidant parameters in PAT closely indicate tumor progression and metastatic status.

## 2. Results

### 2.1. Baseline Characteristics and Comorbidities

This study included 63 participants, comprising the control group (n = 40) and the CRC patients’ group (n = 23). Table 1 shows a summary of the baseline demographic and clinical information. The mean age of the control group was lower than that of the CRC patients’ group (65.85 ± 9.7 years versus 72.9 ± 7.9 years, *p* = 0.0027). The primary participants were men (73.9% in the CRC group and 62.5% in the control group). The average body mass index (BMI) was the same in both groups. The prevalence of hypertension and diabetes mellitus as comorbidities was markedly lower in CRC patients compared to controls (1% vs. 40% and 1% vs. 25%, respectively). Other comorbidities, such as cardiovascular disease and chronic kidney disease, exhibited comparable distributions between the two cohorts.

### 2.2. Plasma MDA and Protein Carbonyl

**Figure 1:**
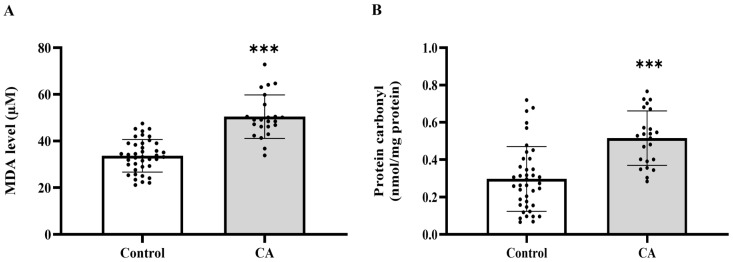
Plasma malondialdehyde (MDA, (**A**)) and protein carbonyl (**B**) of the control and cancer (CA) groups. Results are depicted as mean ± standard error (SEM). Comparison between two groups was performed using Student’s *t*-test. *** *p* < 0.0001. CA: cancer group.

**Figure 2:**
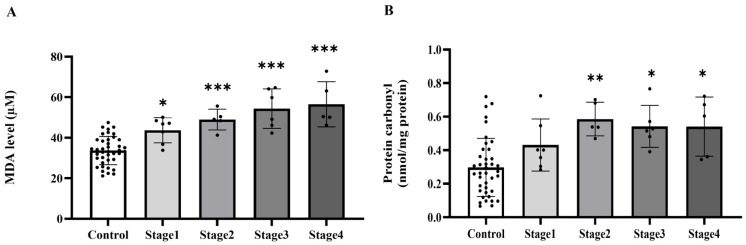
Plasma malondialdehyde (MDA, (**A**)) and protein carbonyl (**B**) of the control group and cancer groups by stage. Results are depicted as mean ± standard error (SEM). *** *p* < 0.0001, ** *p* < 0.001, * *p* < 0.05.

To establish whether systemic oxidative stress is associated with colorectal cancer, we measured the widely used plasma biomarkers MDA and protein carbonyl levels. We found that plasma MDA and protein carbonyl levels were markedly increased in the cancer group relative to the control group (*p* < 0.0001, Figure 1A,B). MDA levels were elevated across all cancer stages when compared to the control group (*p* = 0.0209, 0.0002, 0.0003, 0.0005, respectively). No statistically significant changes were seen across the various cancer stages (*p* > 0.05), suggesting that MDA levels increase from the early stage of cancer without notable variations between stages (Figure 2A). Significant elevations in protein carbonyl levels were noted in stages 2, 3, and 4 relative to the control group (*p* = 0.0041; 0.0101; 0.0221, respectively). Conversely, stage 1 exhibited no significant difference from the control group (*p* = 0.2811), and no statistically significant changes were seen among the cancer stages (*p* > 0.05) (Figure 2B).

### 2.3. The Level of Plasma Catalase and Superoxide Dismutase (SOD) Activities in the Control and Cancer Groups

**Figure 3:**
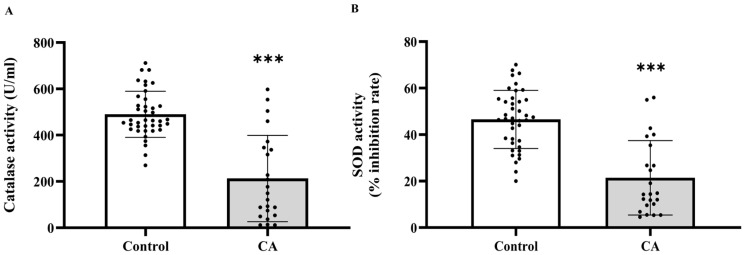
Plasma catalase (**A**) and superoxide dismutase (SOD, (**B**)) activities in plasma of the control and cancer groups. Results are depicted as mean ± standard error (SEM). Comparison between two groups was performed using Student’s *t*-test. *** *p* < 0.0001. CA: cancer group.

**Figure 4:**
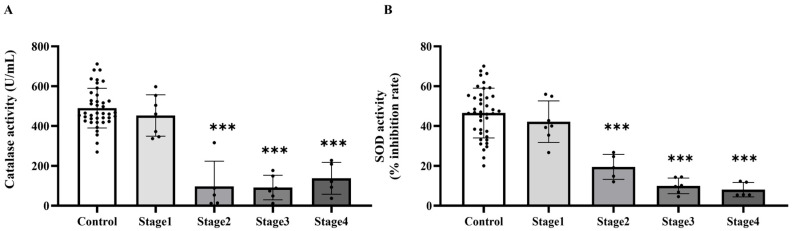
Plasma catalase (**A**) and superoxide dismutase (SOD, (**B**)) activities in plasma of the control and cancer groups by stage. Results are depicted as mean ± standard error (SEM). One-Way ANOVA was used for comparisons between groups. *** *p* < 0.0001.

Based on the increased levels of systemic oxidative stress indicators, we subsequently evaluated whether the activities of essential antioxidant enzymes, catalase and superoxide dismutase (SOD), were similarly modified in plasma. The investigation demonstrated that the activities of both catalase (Figure 3A) and SOD (Figure 3B) were markedly diminished in the cancer cohort relative to the control cohort (*p* < 0.0001). Catalase activity significantly decreased in cancer stages 2, 3, and 4 compared to the control group (*p* = 0.0001, 0.0001, and 0.0002, respectively), although no significant difference was noted between stage 1 and the control group (*p* > 0.05). Moreover, catalase activity in stages 2, 3, and 4 was markedly diminished compared to Stage 1 (*p* = 0.0006; 0.0002; 0.0033, respectively). Nonetheless, no substantial changes were detected among phases 2, 3, and 4 (*p* > 0.05; Figure 4A). A substantial reduction in SOD activity was noted in stages 2, 3, and 4 when compared to the control group (*p* = 0.0199; <0.0001; <0.0001, respectively), whereas no significant difference was found between stage 1 and the control group (*p* > 0.05). SOD activity in Stages 3 and 4 was markedly diminished compared to Stage 1 (*p* = 0.0005; 0.0004, respectively). However, no substantial differences were seen between stage 2 and the subsequent stages, nor between stages 3 and 4 (*p* > 0.05; Figure 4B).

### 2.4. The Level of Superoxide Production in Peritumoral Adipose Tissue in the Control and Cancer Groups

**Figure 5:**
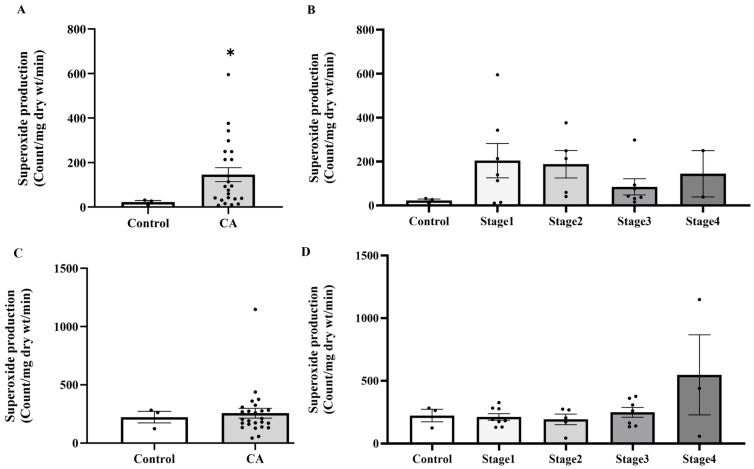
Superoxide production in peritumoral adipose tissue (**A**) and colonic tissue (**C**) in the control and cancer groups and in control and cancer groups by stage (**B**,**D**). Results are depicted as mean ± standard error (SEM). Comparison between two groups was performed using Student’s *t*-test, while One-Way ANOVA was used for comparisons between three or more groups. * *p* < 0.05. CA: cancer group.

Building on evidence of systemic oxidative imbalance, we subsequently examined whether analogous alterations occur locally in PAT, which is a component of the tumor microenvironment. In PAT, a significant increase in superoxide production was observed in the samples isolated from CA groups compared to the normal subjects, as shown in Figure 5A (*p* = 0.00415). However, when analyzed by cancer stage, no statistically significant difference was seen across groups, as shown in Figure 5B (*p* > 0.05). However, there was no significant difference in superoxide production of colonic tissue among control and CA groups, as shown in Figure 5C,D.

### 2.5. The Level of MDA and Protein Carbonyl in Peritumoral Adipose Tissue

**Figure 6:**
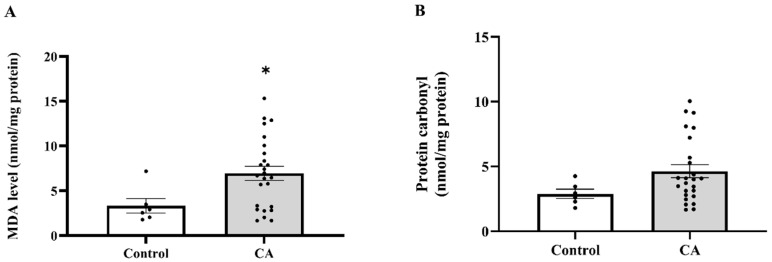
Malondialdehyde (MDA; (**A**)) and protein carbonyl (**B**) in peritumoral adipose tissue of the control and cancer groups. Results are depicted as mean ± standard error (SEM). Comparison between the two groups was performed using Student’s *t*-test. * *p* < 0.05. CA: cancer group.

**Figure 7:**
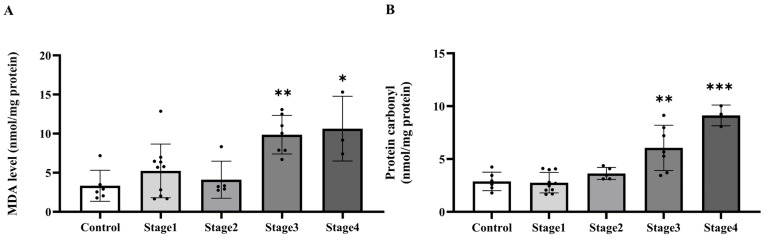
Malondialdehyde (MDA; (**A**)) and protein carbonyl (**B**) in tumoral adipose tissue of the control and cancer (CA) group by stage. Results are depicted as mean ± standard error (SEM). One-Way ANOVA was used for comparisons between groups. *** *p* < 0.0001, ** *p* < 0.001, * *p* < 0.05.

After identifying enhanced superoxide levels in PAT, we evaluated whether subsequent oxidative damage, shown by MDA and protein carbonyl concentrations, was also increased. We found that the tissue MDA levels were elevated in the cancer group relative to the normal subjects (*p* = 0.0376; Figure 6A). Nonetheless, there was no notable difference in carbonyl protein levels when compared to the control group (*p* = 0.1115; Figure 6B). MDA levels in stages 3 and 4 were considerably higher than in the normal subjects (*p* = 0.0035; *p* = 0.0116, respectively), although no significant changes were seen in stages 1 and 2 (*p* > 0.05). Moreover, substantial changes were seen between stage 1 and stage 3 (*p* = 0.0251), as well as between stage 2 and stages 3 and 4 (*p* = 0.0177; *p* = 0.0361, respectively). No additional stage-wise comparisons exhibited statistically significant differences (*p* > 0.05; Figure 7A). Protein carbonyl levels exhibited substantial elevations in stages 3 and 4 relative to the normal subjects (*p* = 0.0013; *p* < 0.0001, respectively), but stages 1 and 2 shown no significant differences from controls (*p* > 0.05). Furthermore, stage-wise analysis demonstrated notable disparities, especially between stage 1 and stages 3 and 4 (*p* = 0.0002; *p* < 0.0001, respectively), as well as between stage 2 and stages 3 and 4 (*p* = 0.0265; *p* < 0.0001, respectively; Figure 7B). These data indicate that protein carbonyl levels generally rise gradually with the advancement of cancer stages.

### 2.6. The Level of Catalase and SOD Activities in Peritumoral Adipose Tissue in the Control and Cancer Groups

**Figure 8:**
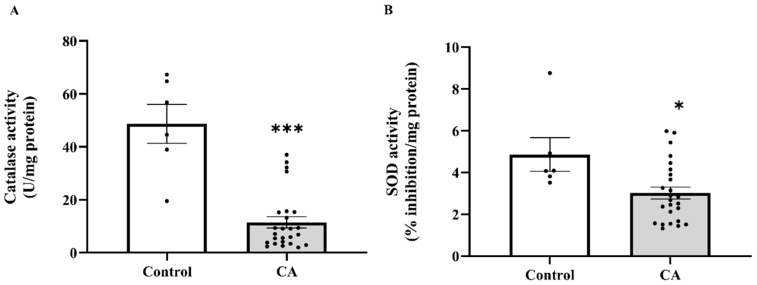
Catalase (**A**) and superoxide dismutase (SOD, (**B**)) activities in peritumoral adipose tissue of the control and cancer groups. Data are expressed as mean ± standard error (SEM). Comparison between two groups was performed using Student’s *t*-test *** *p* < 0.0001, * *p* < 0.05. CA: cancer group.

**Figure 9:**
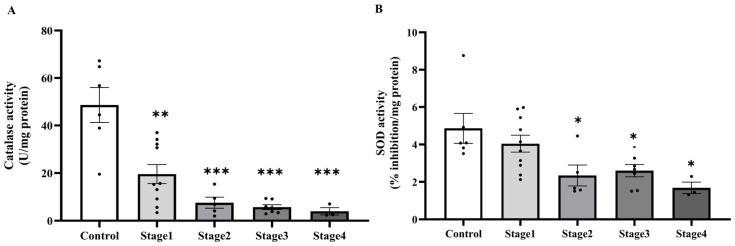
Catalase (**A**) and superoxide dismutase (SOD, (**B**)) activities in peritumoral adipose tissue of the control and cancer groups by stage. Results are depicted as mean ± standard error (SEM). One-Way ANOVA was used for comparisons between groups. *** *p* < 0.0001, ** *p* < 0.001, * *p* < 0.05.

In the context of the increasing oxidative damage markers in PAT, we subsequently assessed local antioxidant capability by measuring catalase and SOD activity in PAT. Consistent with the systemic findings, catalase activity was markedly decreased in PAT of cancer patients compared to the control group (*p* < 0.0001; Figure 8A). Likewise, a significant reduction in SOD activity was observed in the cancer group (*p* = 0.0130; Figure 8B). In addition, stage-specific analysis further indicated that catalase activity was significantly lower across all cancer stages relative to the control group, with statistical significance noted in stage 1 (*p* = 0.0003), stage 2, stage 3, and stage 4 (*p* < 0.0001). However, no significant differences were observed among the cancer stages themselves (*p* > 0.05; Figure 9A). Conversely, SOD activity showed a stage-dependent decline, with significant reductions detected in stage 2 (*p* = 0.0400), stage 3 (*p* = 0.0465), and stage 4 (*p* = 0.0231) compared to the control. In contrast, SOD activity in stage 1 was not significantly different from the control group (*p* = 0.7759), and no significant pairwise differences were observed among the cancer stages (*p* > 0.05; Figure 9B).

### 2.7. The Level Nrf2 and HO-1 Protein Expressions in Peritumoral Adipose Tissue of the Control and Cancer Groups

**Figure 10:**
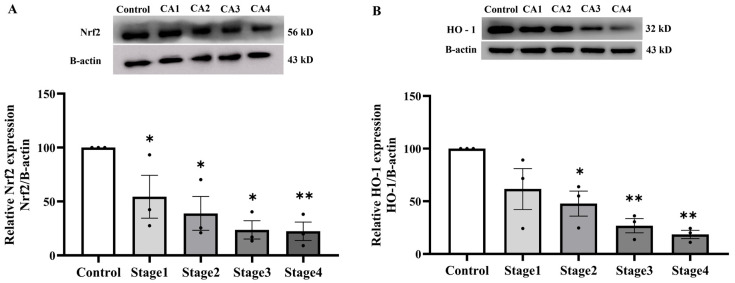
Nrf2 (**A**) and HO-1 (**B**) protein expressions in peritumoral adipose tissue of the control and cancer groups by stage (n = 3/group). Results are depicted as mean ± standard error (SEM). One-Way ANOVA was used for comparisons between groups. ** *p* < 0.001, * *p* < 0.05. CA: cancer group.

To elucidate the mechanisms underlying the antioxidant enzyme deficit in PAT, we subsequently assessed the expression of critical oxidative stress-responsive proteins Nrf2 and HO-1, which regulate cellular antioxidant responses. A marked decline in Nrf2 protein expression has been seen in PAT throughout all cancer stages when compared to normal subjects (*p* < 0.005; Figure 10A). Notably, HO-1 protein expression reduced in cancer stages 2, 3, and 4, but not in stage 1, as seen in Figure 10B.

### 2.8. The Correlation Between Carcinoembryonic Antigen (CEA) Levels and Oxidative Stress Biomarkers in the Plasma and Peritumoral Adipose Tissue

**Figure 11:**
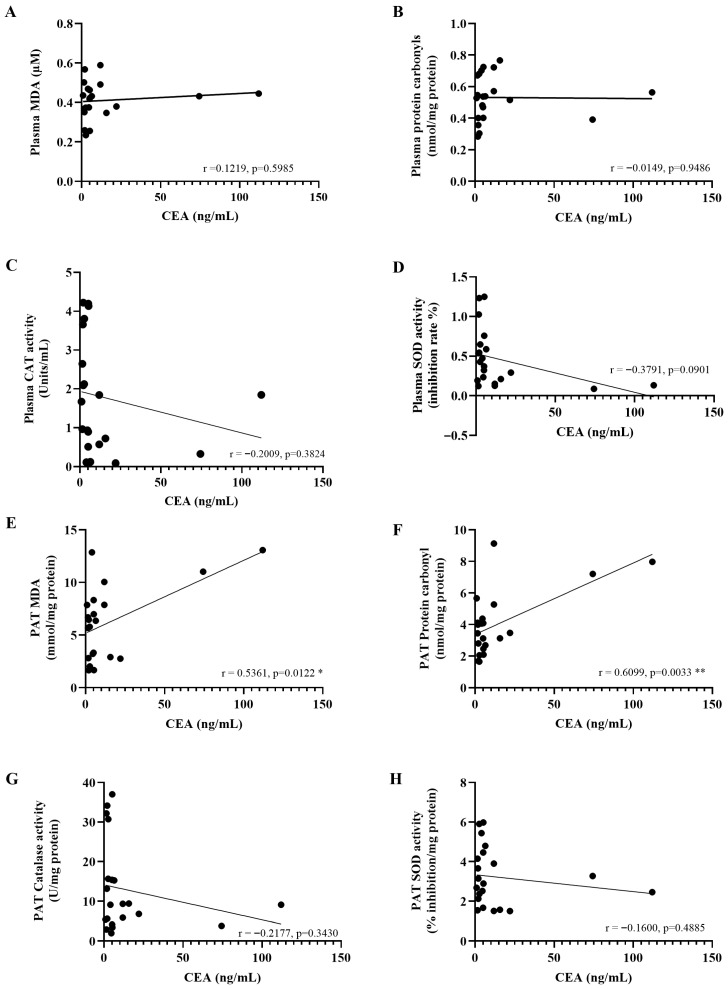
The correlation between carcinoembryonic antigen (CEA) levels and oxidative stress biomarkers in the plasma (**A**–**D**) and peritumoral adipose tissue (**E**–**H**). Pearson’s correlation test was employed to assess the relationships between variables. * *p* < 0.05; ** *p* < 0.01. PAT; Peritumoral adipose tissue.

After identifying a pattern of local and systemic oxidative imbalance, we investigated the possible clinical implications by examining correlations between a tumor marker (CEA) and oxidative stress indicators. A significant positive correlation between CEA levels and the concentrations of MDA (r = 0.5361, *p* = 0.0122) and protein carbonyls (r = 0.6099, *p* = 0.0033) was found in PAT. However, no significant correlations were found between CEA levels and plasma oxidative stress markers (Figure 11A–D), including MDA (r = 0.1219, *p* = 0.5985), protein carbonyls (r = −0.0149, *p* = 0.9486), catalase (r = −0.2009, *p* = 0.3824), and SOD (r = −0.3791, *p* = 0.0901). Similarly, no significant associations were observed between CEA levels and the levels of catalase (r = −0.2177, *p* = 0.3430) or SOD (r = −0.1600, *p* = 0.4885) in PAT (Figure 11E–H).

## 3. Discussion

This study found a marked elevation of multiple oxidative stress markers, including superoxide production, MDA, and protein carbonyl, alongside a reduction in antioxidant defense enzymes such as superoxide dismutase and catalase, in people diagnosed with CRC. These biomarkers exhibited increasing alterations associated with advancing stages of CRC. Additionally, MDA and protein carbonyl exhibited in PAT exhibited a positive correlation with serum CEA. The expression of Nrf2/HO-1 proteins in PAT was reduced in CRC.

Our findings indicate that patients with CRC exhibited elevated MDA and protein carbonyl concentrations, coupled with diminished catalase and superoxide dismutase activities, signifying oxidative stress in this population. Our findings aligned with prior research indicating that lipid peroxidation and protein oxidation were heightened in cancer patients relative to healthy individuals [15,19]. Indeed, oxidative stress is important to the development and progression of cancer since it promotes DNA mutations, DNA damage, genomic instability, and cell growth [15,20]. Under physiological conditions, ROS is eliminated by endogenous antioxidant enzymes. In this study, key antioxidant enzymes, including catalase and superoxide dismutase, which neutralize reactive oxygen species, were diminished in colorectal cancer patients compared to healthy individuals. The findings concurred with a previous study that demonstrated reduced activity of the antioxidant enzymes catalase and superoxide dismutase in colorectal cancer, indicating a compromised antioxidant defense mechanism [21]. In contrast, several studies demonstrate a significant increase in SOD [22] alongside a notable decrease in CAT [23], suggesting a complex and possibly paradoxical reaction to the redox imbalance associated with CRC. Reactive oxygen species (ROS) frequently originate from the mitochondrial respiratory chain. Their neutralization is managed sequentially, first by SOD converting them into hydrogen peroxide (H_2_O_2_), and then by catalase breaking down the H_2_O_2_ [24]. The decline in antioxidant enzyme activity noted in cancer may be ascribed to various processes, including particular genetic variants of SOD and catalase [25], tumor-specific adaptations [26], and the effects of chemotherapy [27].

A novel aspect of our work is the examination of PAT biomarkers and their association with systemic tumor indicators. PAT play a critical role in the cancer microenvironment PAT may exacerbate the pro-oxidant microenvironment through the secretion of inflammatory mediators that synergistically amplify both inflammatory cascades and oxidative stress pathways, consequently promoting neoplastic cell proliferation [17]. We discovered that oxidative stress markers in circulation are consistent with those in peri-tumor adipose tissue, suggesting a potential connection between local tissue biology and circulating biomarkers. In CRC, increases in superoxide production, MDA and protein carbonyl as well as low activities of endogenous antioxidant enzymes in the PAT were observed, indicating oxidative stress and cellular damage [20,28]. There was no difference in superoxide production in adjacent normal colonic tissue between the control and CRC groups, highlighting the significant function of PAT in CRC. While certain prior studies have examined the role of adipose tissue inflammation within the tumor microenvironment [29], a limited study has specifically explored oxidative stress indicators in peri-tumoral adipose tissue in relation to proven tumor biomarkers such as CEA. Our findings suggest that oxidative stress in the PAT microenvironment may influence or indicate tumor growth and systemic tumor burden. Future research and mechanistic investigations employing adipocyte–tumor co-culture models are necessary to determine if PAT oxidative stress serves as a trigger for carcinogenesis through paracrine signaling or only signifies an exacerbation of tumor burden.

Our results further extend a progressive oxidative–antioxidative imbalance across CRC stages since these alterations intensified with advancing CRC stage, suggesting a stage-dependent dysregulation of redox homeostasis in the circulation and tissue. Our findings corroborate previous studies that serum levels of MDA, a product of lipid peroxidation, were incrementally elevated in patients with CRC, attaining peak values in the fourth stage of the disease [30]. Elevated protein carbonyl levels in plasma correlated with cancer stage and extended fibrin clot lysis time in lung cancer [31]. We initially established the correlation between colorectal cancer stage and oxidative stress markers as well as endogenous antioxidant enzymes in adipose tissue around the tumor, highlighting the significant influence of the oxidative–antioxidant balance within the circulation and cancer microenvironment related to the severity of colorectal cancer staging.

Moreover, another novel finding from our study is the decreased Nrf2 and HO-1 expression in PAT of CRC cases that was associated with the activities of endogenous antioxidant enzymes. Although previous research has implicated Nrf2 pathway activation in the adaptive response to oxidative stress in CRC tumor cells [32], our data highlight a significant downregulation in adjacent adipose tissue, an area less explored. The role of the Nrf2/HO-1 axis in PAT suggests a broader systemic adjacent tissue response to CRC-driven oxidative stress, potentially relating to tumor progression. This is the inaugural research indicating the downregulation of Nrf2/HO-1 pathway proteins in PAT in CRC. These could indicate the different defense mechanisms to oxidative stress in cancer cells and adjacent tissue, PAT.

## 4. Materials and Methods

### 4.1. Study Population

The present work is a cross-sectional study that enrolled 63 individuals, comprising 40 control subjects who underwent annual colonoscopy checkups and 23 patients diagnosed with colorectal cancer who were undergoing treatment at Srinagarind Hospital, Faculty of Medicine, Khon Kaen University, Khon Kaen, Thailand. Inclusion criteria: All participants who provided written informed consent before enrollment were included in this study. Participants were diagnosed and categorized into five groups based on disease staging: the control group consisted of healthy individuals undergoing colonoscopy check-up; Stage 1 included patients with tumor confined to the intestinal wall; Stage 2 comprised patients whose tumor infiltrates beyond the gut wall without lymphatic node involvement; Stage 3 consisted of patients with tumor spread to regional lymph nodes; and Stage 4 included patients with tumor metastasis to distant organs such as the liver and lungs. Exclusion criteria: participants had a history of active or untreated diseases, including autoimmune disease, infectious disease, or individuals who did not provide or were unable to provide written informed consent. This study used leftover specimens, including plasma and PAT from standard laboratory procedures at Srinagarind Hospital, Faculty of Medicine, Khon Kaen University, Khon Kaen, Thailand. The study protocol was approved by the Khon Kaen University Ethics Committee for Human Research (HE641195) and conducted in accordance with the Declaration of Helsinki and relevant local regulations.

### 4.2. Sample Collection

A leftover plasma specimen from a routine laboratory analysis was collected from the clinical laboratory, Srinagarind Hospital, Faculty of Medicine, Khon Kaen University, Khon Kaen, Thailand. Storage of samples at −20 °C was performed until analysis. Plasma samples were used to determine levels of carcinoembryonic antigen (CEA), oxidative stress markers including MDA and protein carbonyl, as well as the activities of antioxidant enzymes such as SOD and catalase. PAT samples were collected from colorectal cancer patients during surgical resection for a routine pathological analysis. Leftover tissue fragments (approximately 20 mg) were obtained from areas adjacent to the tumor mass. The samples were promptly stored on ice and washed with a saline solution to remove remaining blood and other impurities. The tissue samples were thereafter preserved at −20 °C. 

### 4.3. Measurement of Superoxide Production, Malondialdehyde (MDA) Level in Plasma and Peritumoral Adipose Tissue

Superoxide production in the PAT was measured based on the lucigenin-enhanced chemiluminescence protocol, following a previous study [33]. The data are presented as relative light unit counts per minute per gram of dry tissue weight. MDA concentrations in plasma and PAT were evaluated according to the established procedure [33]. In summary, plasma (150 μL) was combined with a solution containing TCA (10%), EDTA (5 mmol/L, 8%) SDS, and BHT (0.5 μg/mL), allowed to stand at room temperature for 10 min, after which 0.6% TBA was introduced. The material underwent boiling for 30 min before to centrifugation for 10 min at 1000× *g*. The supernatant was obtained, and the absorbance was measured at 532 nm utilizing a spectrophotometer. The data were derived from a standard curve established using 1,1,3,3-tetraethoxypropane. The concentration of MDA was expressed as nmol/mg protein.

### 4.4. Measurement of Carbonyl Protein Levels in Plasma and Peritumoral Adipose Tissue

The concentration of protein carbonyls in plasma and PAT was detected using 2,4-dinitrophenylhydrazine (DNPH) derivatization, as previously described [34]. Briefly, the protein content in plasma and PAT samples from cancer patients was measured following the protocol adapted from [34]. The samples were diluted and then incubated with the Bradford reagent. The absorbance of the mixture was measured at 595 nm using a microplate reader. The amount of protein content was calculated using a standard curve created from bovine serum albumin (BSA). The supernatant was then spectrophotometrically analyzed at 370 nm, and the level of carbonyl protein was presented as nmol/mg protein.

### 4.5. Measurement of Catalase Activity in Plasma and Peritumoral Adipose Tissue

Catalase activity in plasma and PAT was assessed following the previously described protocol [33,35]. In short, 20 µL of the sample was mixed with 65 μmol/mL H_2_O_2_ in a 60 mmol/L PBS (pH 7.4) solution and kept at 37 °C for 1 min. Thereafter, 100 µL of ammonium molybdate was added to stop the reaction, and the absorbance of the mixture was measured at 405 nm with a spectrophotometer. The amount of catalase activity was determined using a standard curve from bovine liver (Sigma-Aldrich, St. Louis, MO, USA) and reported as U/mg protein.

### 4.6. Measurement of Superoxide Dismutase (SOD) Activity in Plasma and Peritumoral Adipose Tissue

SOD activity in plasma and PAT was assessed using the SOD Assay Kit (Dojindo Laboratories, Kumamoto, Japan). In summary, 20 µL of the sample was poured into the well and mixed with the working solution, dilution buffer, and enzyme solution, then maintained at 37 °C for 20 min. Absorbance was quantified at 450 nm by spectrophotometry. The SOD activity was expressed as the percentage inhibition rate per milligram of protein.

### 4.7. Measurement of Nuclear Factor Erythroid 2-Related Factor 2 (Nrf2) and Heme Oxygenase-1 (HO-1) Protein Expression in Peritumoral Adipose Tissue

The protein extraction process from PAT was adapted from the method of Yu A. An and Philipp E. Scherer [36]. Initially, RIPA buffer without Triton X-100 was used to lyse fat cells and release proteins. The sample was then centrifuged at 6000× *g* for 15 min at 4 °C to remove lipid contamination. The lipid layer was separated from the supernatant, which contains the desired protein extract. Triton X-100 was added to further lyse the cells, followed by high-speed centrifugation at 12,000× *g* for 15 min to obtain fat-free protein. The supernatant was collected, and protein content was quantified using the BCA protein assay kit, facilitating precise measurement for subsequent Western blot analysis. The protein from PAT (40–60 µg) was separated by 10–12% SDS-PAGE gels. Primary antibodies used included Nrf-2 (Santa Cruz Biotechnology, Heidelberg, Germany; 1:1000) and HO-1 (Santa Cruz Biotechnology, Heidelberg, Germany; 1:500), which were incubated overnight at 4 °C. After washing, secondary antibodies (anti-mouse IgG peroxidase conjugated) and (goat anti-rabbit IgG peroxidase conjugated) were applied for 1 h at room temperature. Thereafter, the membranes were washed and incubated with enhanced chemiluminescence reagents (ECL Prime, Amersham Bioscience, Little Chalfont, Buckinghamshire, UK). The intensity of the bands was detected by the ChemiDoc XRS+ imaging system (Bio-Rad, Hercules, CA, USA). The expression levels of Nfr-2 and HO-1 were normalized to β-actin (Santa Cruz Biotechnology, Heidelberg, Germany; final dilution 1:4000).

### 4.8. Statistical Analysis

Statistical analysis was performed using Graph-Pad Prism software version 10.4.2 (GraphPad, San Diego, CA, USA). The Shapiro–Wilk test was used to assess data normality. Comparison between two groups was performed using Student’s *t*-test, while One-Way ANOVA was used for comparisons between three or more groups. Pearson’s correlation test was employed to assess the relationships between variables. Differences were considered significant when *p* < 0.05.

## 5. Conclusions

Our findings not only support the established role of oxidative stress and disrupted antioxidant defenses in CRC but also emphasize the significance of the tumor–adipose microenvironment, particularly PAT, in mediating redox changes that are linked to disease severity and systemic tumor markers. This provides new insights into the pathophysiology of CRC and highlights potential redox biomarkers and therapeutic targets within the tumor microenvironment.

## Figures and Tables

**Table 1:** Baseline characteristics and comorbidities of the study population.

Variable	Control (n = 40)	CRC Patients (n = 23)	*p* Value
**Demographic and anthropometric data**			
Age (years), mean ± SD	65.85 ± 9.7	72.9 ± 7.9	0.0027 ^†^
Male sex, n (%)	25 (62.5)	17 (73.9)	0.41
BMI (kg/m^2^), mean ± SD	24.08 ± 3.9	23.7 ± 3.7	0.73
**Comorbidities, n (%)**			
Diabetes Mellitus	10 (25.0)	1 (4.3)	0.044 ^†^
Hypertension	16 (40.0)	1 (4.3)	<0.0025 ^†^
Dyslipidemia	12 (30.0)	8 (34.7)	0.78 ^†^
Obesity	15 (37.5)	10 (43.5)	0.79 ^†^
Cardiovascular disease	5 (12.5)	0 (0.0)	0.15 ^†^
Chronic kidney disease	4 (10.0)	1 (4.3)	0.65 ^†^

^†^ *p*-values calculated by Fisher’s exact test.

## Data Availability

The original contributions presented in this study are included in the article. Further inquiries can be directed to the corresponding author.

## Abbreviations

The following abbreviations are used in this manuscript:

PAT	Peritumoral adipose tissue
SOD	Superoxide dismutase
MDA	Malondialdehyde
CRC	Colorectal cancer
Nrf2	Nuclear factor erythroid 2-related factor 2
HO-1	Heme oxygenase-1
CEA	Carcinoembryonic antigen
